# Stratification of Patients with Coronary Artery Disease by Circulating Cytokines Profile: A Pilot Study

**DOI:** 10.3390/jcm12206649

**Published:** 2023-10-20

**Authors:** Concetta Iside, Ornella Affinito, Bruna Punzo, Marco Salvatore, Peppino Mirabelli, Carlo Cavaliere, Monica Franzese

**Affiliations:** 1 IRCCS SYNLAB SDN, Via Emanuele Gianturco, 113, 80143 Naples, Italy; 2 Department of Pediatric Hemato-Oncology, Santobono-Pausilipon Children’s Hospital, AORN, 80122 Naples, Italy

**Keywords:** coronary artery disease, cardiovascular disease, diagnostic markers, inflammation cytokines

## Abstract

Coronary artery disease (CAD) is a long-term inflammatory process, with atherosclerosis as its underlying pathophysiological mechanism. Endothelial dysfunction is the first step towards atherosclerosis, where damaged endothelial cells release large amounts of pro-inflammatory cytokines and mediators, thus promoting vascular inflammation and disease progression. However, the correlation between serum cytokines and CAD severity remains to be defined. Serum samples from patients performing cardiac computed tomography for suspected CAD (*n* = 75) were analyzed with a multiplex bead-based immunoassay panel for simultaneous assessment of the concentration of 11 cytokines using flow cytometric technology. The analysis showed statistically significant increases in sRAGE, CCL2_MCP1, FLT1, and IL6 levels in CAD patients compared with healthy subjects and a gradual increase trend towards a more severe form of the disease for most cytokines (e.g., sCD40L, FLT1, sRAGE, CCL2-MCP1, TNFα). Lastly, we explored the performance of cytokines in predicting the diagnosis of CAD and found that an increase in IL6 levels will increase the odds of being non-obstructive CAD-positive. In contrast, an increase in CCL2-MCP1 or FLT1 levels will increase the probability of being obstructive CAD-positive. These results suggest that the combination of serum cytokines may contribute to the not-invasive stratification risk for patients with suspected CAD.

## 1. Introduction

Coronary artery disease (CAD) is one of the major causes of morbidity and mortality worldwide [1]. Current methods (i.e., clinical risk score systems or plasma biomarkers) still present critical challenges for identifying patients with residual risk for coronary events. Recent clinical evidence has emphasized the importance of assessing coronary architecture through coronary computed tomography angiography (CCTA) in the management of patients with CAD [2].

CAD is a chronic inflammatory disease triggered by atherosclerosis and affects both the media and intimal layer of the coronary arteries [3,4]. The inflammation process plays a key role in the initiation and progression of CAD by the release of proinflammatory cytokines, which in turn induces the activation of inflammatory cells (macrophages and monocytes) [5]. In arterial vessels, HDL and LDL lipoproteins less than 70 nm in diameter can cross the endothelial layer and penetrate the intima of the vessels. The infiltrated lipoproteins can undergo modification by oxidizing agents, proteases, and lipases. These modified lipoproteins can promote inflammatory atherosclerotic lesion formation by activating and recruiting the inflammasome complex and leukocytes. A key role in this process is played by macrophages that take up the modified lipoproteins by promoting foam cell formation. In the initial stages, acute inflammation represents a protective and resolving process driven by anti-inflammatory molecules such as IL-10, which can reduce leukocyte recruitment [6].

CD40L is mainly expressed by activated T cells and platelets, and the plasma levels of soluble CD40L (sCD40L) were increased in patients with acute coronary syndromes [7]. It has been reported that placental-like growth factor (PIGF) and soluble Fms-like tyrosine kinase-1 (sFlt-1) are associated with adverse outcomes in patients with heart failure (HF), and elevated levels of sFlt-1 are associated with adverse outcomes in stable patients with HF [8]. CD4+ cells play a complex role in the inflammatory process of coronary artery diseases, contributing both to chronic inflammation within atherosclerotic plaques and to the overall regulation of the immune response [9]. In individuals with HF, CD4+ T cells can become pathological. Kumar et al. reveal that the expression of tumor necrosis factor (TNF)-α and its receptor, TNFR1, increases in HF-activated CD4+ T cells [10]. Within cardiovascular diseases, coronary artery disease and the underlying atherosclerosis have recently been linked to both systemic and local inflammation. The cell surface receptor for extracellular cyclophilins, the CD147 receptor, also contributes to the pathogenesis of coronary artery disease. Cyclophilins, pro-inflammatory interleukins, and CD147 membrane expression were significantly elevated in patients with coronary artery disease [11].

The multi-ligand receptor for advanced glycation end products (RAGE) is an important mediator of inflammation, while the soluble form of RAGE (sRAGE) serves as a decoy. It has been reported that higher levels of AGEs/sRAGE ratio are associated with CAD in asymptomatic patients [12]. Convincing evidence has shown the association of TNFα, IL-1β, and MCP-1 with CAD pathogenesis [13], while serum IL-6 has been considered a predictive marker for CAD [14]. Most studies have mainly explored the differences in serum cytokine levels between CAD patients with healthy subjects. Only a few studies have evaluated the relationship between cytokine levels and the extent of coronary atherosclerotic plaque burden. Moreover, although CCTA has emerged as a promising, cost-effective, and non-invasive imaging modality for CAD diagnosis, plaque identification, and characterization [15], a limited number of studies have investigated the association of high-risk features assessed by CCTA with circulating biomarkers.

The current study aimed to address the following key objectives: (i) analyze the relationship between serum cytokine levels (sCD40L, FLT1, sRAGE, CCL2-MCP1, TNFα, IL-6, IL-18, IL-10, sST2, PIGF, and LIGHT) and the severity of CAD; (ii) investigate specific cytokines potentially associated with a higher risk of severe coronary artery lesions; (iii) identify potential predictors of severe CAD in individuals with suspected CAD.

## 2. Material and Methods

### 2.1. Study Population

During a period of 13 months (from 1 January 2020 to 1 March 2021), we prospectively enrolled a total of 75 consecutive subjects with right coronary dominance without a previous history of cardiovascular events and clinically referred to our institution for suspected CAD and CCTA assessment. In addition, in order to be included, individuals also had to have a stable sinus rhythm and normal heart chambers, both anatomically and functionally. Patients with left coronary dominance, any kind of complete or partial bundle branch block, cardiomyopathy, aortic diseases, previous percutaneous transluminal coronary angioplasty, and coronary artery bypass grafting were not included in the study population. Demographic characteristics, height, body weight, and presence of cardiovascular risk factors (i.e., predisposition to CAD, hypertension, dyslipidemia, type II diabetes, obesity) were prospectively collected. The study was approved by the Institutional Ethics Committee in accordance with the ethical standard of the Declaration of Helsinki (Protocol 2/19). Written informed consent was obtained from all participants.

### 2.2. Imaging Protocol

All patients underwent CCTA with a 3rd generation Dual Source multidetector CT scanner (Somatom Force, Siemens Healthineers, Forchheim, Germany). The effective temporal resolution of the equipment was 66ms for a single reconstructed axial slice, allowing to obtain perfectly stable and motionless diastolic and systolic datasets, regardless of the heart rate. A prospectively ECG-triggered high-pitch spiral acquisition (FLASH) without a contrast medium was performed for calcium score evaluation (slice thickness of 3 mm, increment of 3 mm, small cardiac FOV). Afterward, patients underwent angiographic CCTA scans with IV contrast material (50 mL@5 mL/s of iodinated contrast agent—Iomeprol 400 mgI/mL, Iomeron 400, Bracco, Milan, Italy—followed by 50 mL@5 mL/s of saline flush); scans were performed with retrospective ECG-gating and with prospective ECG-tube current modulation (window: 25–75% of the RR interval). Automated attenuation-based anatomical tube current (mAs) modulation (CARE Dose4D, Siemens Healthineers) and automated attenuation-based tube voltage (kV) selection functionality (CARE kV, Siemens Healthineers) were used for dose reduction and optimization. Agatston score and stenosis degree for patient stratification were assessed using post-processing (Syngo.Via Software, version n. VB60A_HF04, Siemens Healthineers, Forchheim, Germany).

Based on the outcome of the CCTA investigation, the study population (*n* = 75) was stratified into three groups: controls (CTRL; *n* = 20), obstructive CAD (obCAD; ≥50% stenosis; *n* = 21), non-obstructive CAD (non_obCAD; < 50%; *n* = 34) [16].

### 2.3. Laboratory Methods

Serum cytokine levels were evaluated using a flow cytometry bead-based LEGENDplex Human Vascular Inflammation Panel assay (BioLegend Way, San Diego, CA, USA). Frozen serum samples were analyzed using an 11-plex human vascular inflammation panel to analyze the following cytokines: sCD40L, FLT1, sRAGE, CCL2-MCP1, TNFα, IL-6, IL-18, IL-10, sST2, PIGF, and LIGHT. Samples were pre-diluted 1:2 and the assay was run according to the manufacturer’s instructions. Sample data were collected using the Cytoflex V2-B4-R2 (Beckman-Coulter, Brea, CA, USA). Cytokine concentrations for each sample were determined from extrapolation from standard curves using the LEGENDplex Data Analysis Software according to the manufacturer’s protocol.

### 2.4. Data Processing

Cytokine values under the analytical detection limit were considered as not available (NA). For each patient and for each cytokine, values were averaged between the two replicates. After processing procedures, for each patient’s group and each cytokine, the sample size is reported in Table 1.

### 2.5. Statistical Analysis

Continuous variables are expressed as mean ± standard deviation (SD) if normally distributed and as median and range if not. Categorical variables are expressed as absolute values. The normality assumption for continuous variables was assessed using the Shapiro–Wilk test. The association between categorical variables was assessed using Pearson’s chi-squared test or Fisher’s exact test. For comparisons between two groups (CTRL and CAD), a two-tailed Wilcoxon rank sum test was used. Multiple comparisons among the three groups (CTRL, obCAD, and non_obCAD) were performed by using the Kruskal–Wallis test with Dunn’s post hoc test and Bonferroni correction. Correlation analyses between quantitative variables were determined with Spearman’s correlation test. The predictive accuracy of the single markers in discriminating two groups (CTRL vs. obCAD and CTRL vs. non_obCAD) was measured using the area under the receiver operating characteristic (ROC) curve (AUC). The optimal cut-off values were chosen by maximizing the Youden’s index. The ability of quantitative variables to predict the outcome (diagnosis) was assessed with logistic regression analysis. *p*-value ≤ 0.05 was considered statistically significant. All analyses were performed with R 4.2.1 (https://www.r-project.org/, accessed on 28 September 2022).

## 3. Results

### 3.1. Clinical Features of the Study Population

Individuals enrolled in the study (*n* = 75) were grouped as controls (CTRL; *n* = 20), obstructive CAD (obCAD; *n* = 21), and non-obstructive CAD (non_obCAD; *n* = 34). For each group, clinical features are listed in Table 2, along with the corresponding measures of central tendency and dispersion.

Age and Agatston variables were significantly higher in obCAD and non_obCAD compared to CTRL and significantly higher in obCAD compared to non_obCAD, as revealed by CCTA. No differences were found among the three groups for other quantitative variables (Table 2). There were also statistically significant associations with hypertension. No statistical association was found with other categorical variables (Table 2). In addition, the Agatston score shows a significant predictive effect in discriminating obCAD from non_obCAD (OR = 1.004, 95% CI = 1.002–1.007, *p*-value = 0.002) [17].

The main coronary vessel affected was IVA for both non_obCAD and obCAD groups. Plaque characteristics have calcified components in 52.38% of cases and prevalent non-calcified plaques in 28.57%. For the remaining cases, the mixed component (calcified and noncalcified) is balanced with a similar distribution within both injured groups.

### 3.2. Evaluation of Inflammation Levels in Groups of Patients

**Figure 1:**
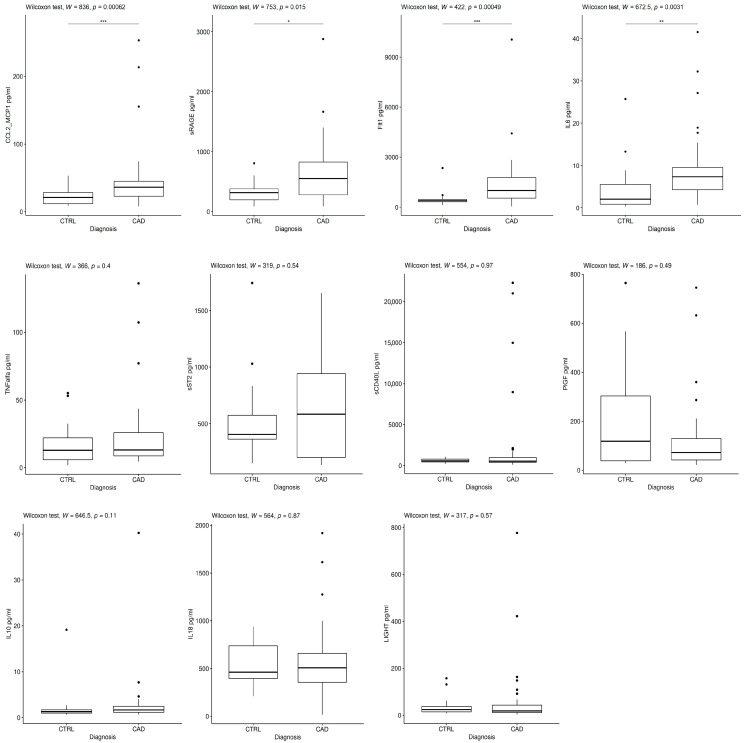
Distribution of different cytokine serum concentrations in CTRL and CAD groups. For each cytokine, boxplots show the concentration in CTRL and CAD. Statistical differences were calculated by using the Wilcoxon test (* *p* ≤ 0.05; ** *p* ≤ 0.01; *** *p* ≤ 0.001).

Serum samples of the two groups of patients (CTRL and CAD) were screened for the concentration of a panel of cytokines. The analysis revealed that CAD patients showed a significant statistical increase in sRAGE, CCL2_MCP1, FLT1, and IL6 levels (1.9, 2.0, 3.1, and 2.0-fold, respectively) compared to controls. No significant difference was found between the two groups for the other cytokines (Figure 1). Thus, higher levels of specific cytokines characterize the serum of CAD subjects.

**Figure 2:**
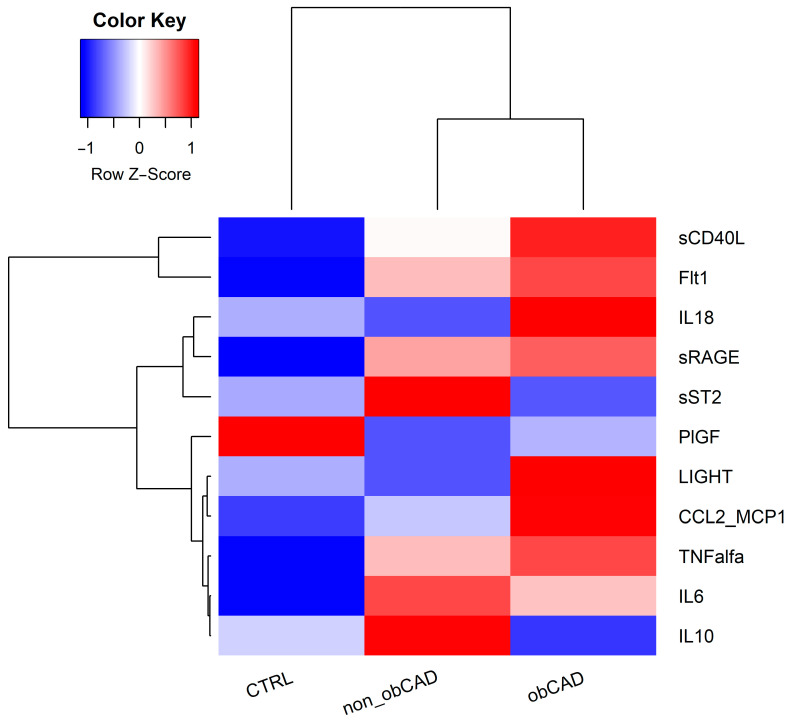
Cytokine serum concentrations in CTRL, non_obCAD, and obCAD. The heatmap shows the mean concentration of each cytokine in the 3 groups. Levels of each cytokine (rows) were averaged within the pre-classified group (column). Unsupervised hierarchical clustering is also reported between cytokines and between groups.

Most of the cytokines (i.e., sCD40L, FLT1, sRAGE, CCL2-MCP1, TNFα) showed a gradually increasing trend going towards a more severe form of the disease (i.e., obCAD). Only the PIGF cytokine levels showed a decreasing trend from CTRL to obCAD. Other cytokines (i.e., sST2, IL6, IL10, IL18, LIGHT) did not show a well-defined trend (Figure 2). A one-way ANOVA was performed to examine the differences in the cytokines’ levels in 3 groups of patients (CTRL, obCAD, and non_obCAD) (Table 3). Except for sRAGE, we confirmed the statistical significance for CCL2_MCP1, FLT1, and IL6 (Kruskall–Wallis test; *p*-values = 0.001, 0.003, 0.006, respectively). Specifically, non_obCAD showed an increase in CCL2_MCP1 and IL6 levels (1.7 and 1.35-fold, respectively) compared to controls, while obCAD showed an increase in CCL2_MCP1 and FLT1 levels (1.05 and 0.84-fold, respectively) compared to controls. No significant difference was found between non_obCAD and obCAD groups.

### 3.3. Relationship between Clinical Features and Cytokines Concentration

**Figure 3:**
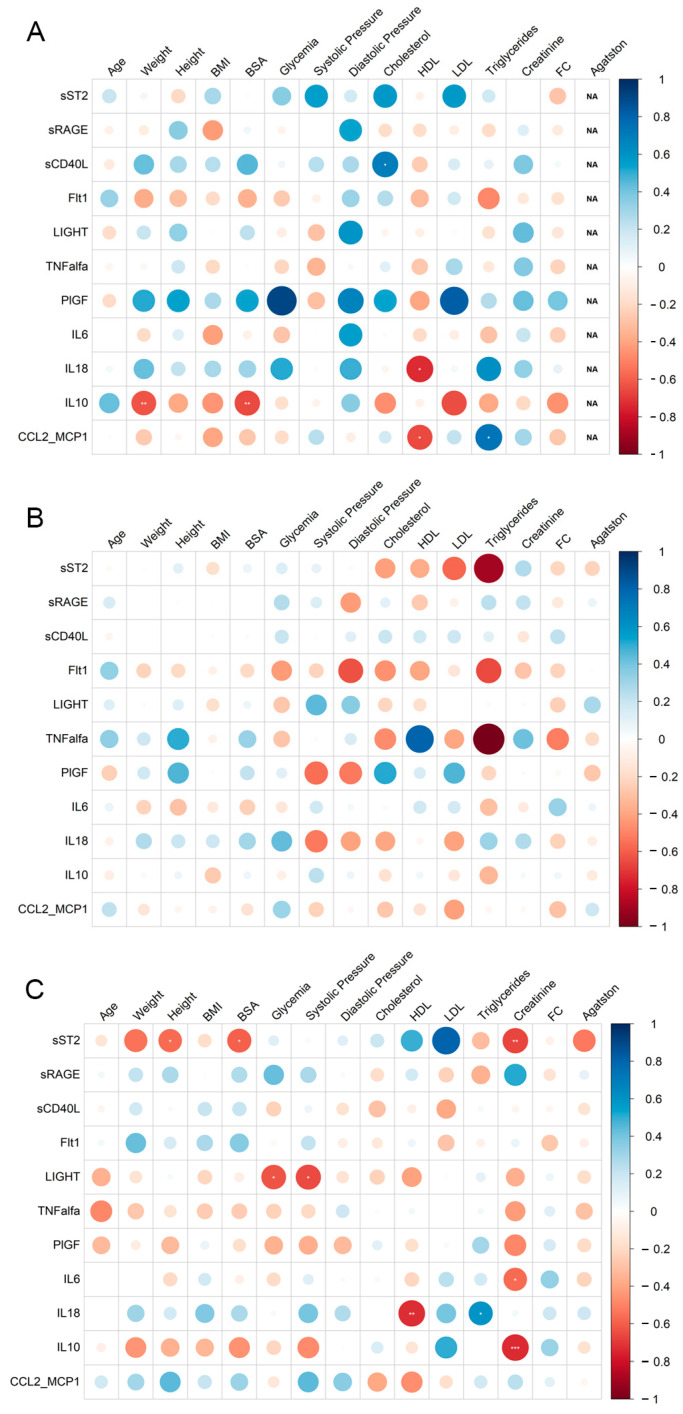
Correlation plots. Correlograms show the Spearman’s correlation between cytokine levels and clinical variables in (**A**) CTRL subjects, (**B**) non_obCAD, and (**C**) obCAD patients. The circle size is scaled by the correlation coefficient. Blue and red colors designate, respectively, the positive and negative correlations (* *p* ≤ 0.05; ** *p* ≤ 0.01; *** *p* ≤ 0.001).

The association between clinical features and cytokine concentration was estimated using Spearman correlation (Figure 3, Table 4). We only considered correlations with |*r*| ≥ 0.6, *p*-value ≤ 0.05 statistically significant and estimated in at least 50% of samples of the belonging group. In the obCAD group, we observed significant negative correlations between sST2 and height, BSA and creatinine, IL10 and creatinine, IL18 and HDL, IL6 and creatinine, and LIGHT and glycemia and systolic pressure. However, a significant negative correlation between IL18 and HDL was also observed in the CTRL group. Moreover, a significant positive correlation also existed between IL18 and triglycerides. No significant correlation was found in the non_obCAD group (Figure 3, Table 4).

### 3.4. Diagnostic Performance of the Selected Cytokines

We estimated the diagnostic performance of cytokines (sRAGE, CCL2_MCP1, FLT1, and IL6) showing a statistically significant concentration between CTRL vs. obCAD and CTRL vs. non_obCAD.

**Figure 4:**
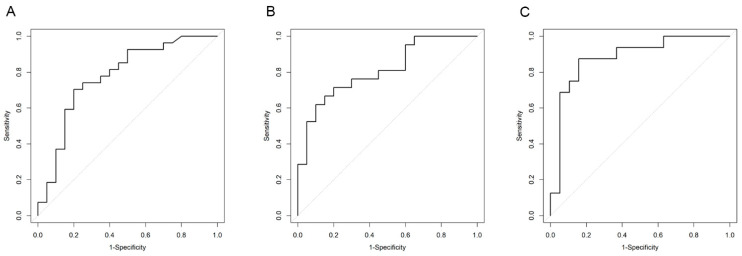
Receiver operating characteristic (ROC) curve for (**A**) IL6 in non_obCAD, (**B**) CCL2-MCP1, and (**C**) Flt1 in obCAD. The diagonal represents the reference line. The solid line represents the ROC curve of the cytokine.

Receiver operating characteristic (ROC) curve analysis confirmed the prognostic role of IL6 in discriminating CTRL vs. non_obCAD (AUC = 0.77; 95% CI: 0.63–0.92) with a cut-off value of 5.91 pg/mL (sensitivity: 0.70 and specificity: 0.80) (Figure 4a).

CCL2_MCP1 and FLT1 showed a significant discriminatory accuracy between CTRL vs. obCAD. Specifically, the AUC for CCL2-MCP1 was 0.81 (95% CI: 0.68–0.94) with a cut-off value of 34.32 pg/mL (sensitivity: 0.90 and specificity: 0.62) (Figure 4b); the AUC for FLT1 was 0.89 (95% CI: 0.76–1.00) with a cut-off value of 563.50 pg/mL (sensitivity: 0.87 and specificity: 0.84) (Figure 4c). No significant prognostic value was observed for sRAGE.

Using univariate logistic regression analysis, we explored the performance of the above cytokines in predicting CAD diagnosis and we found that an increase in IL6 levels will increase the odds of being non_obCAD-positive, while an increase in CCL2-MCP1 or FLT1 levels will increase the odds of being obCAD-positive (Table 5).

## 4. Discussion

CAD stands as the leading cause of mortality worldwide. The severity of CAD closely intertwines with clinical outcomes, as patients with more advanced stages face heightened risks of cardiovascular death and myocardial infarction. Consequently, it becomes paramount to stratify and identify individuals with severe CAD, as this knowledge can significantly enhance CAD prevention, diagnosis, and treatment strategies.

In this context, the technological development of 3rd generation Dual Source multidetector CT scanners has fostered the possibilities to identify and characterize CAD also in complex cardiovascular anatomy and non-compliant patients, in fact promoting CCTA as a screening method towards more invasive procedures (e.g., coronary angiography) [18]. The ‘napkin ring’ sign, positive parietal remodeling, low density of the non-calcific component, and low or no plaque calcification are among the criteria of plaque vulnerability that are now feasible and thought to be at least somewhat trustworthy [19]. Although several of these characteristics are more challenging to replicate and resolve, current developments in photon-counting CT technology promise to overcome any resolution and plaque tissue characterization constraints [20]. In the meantime, peripheral blood biomarkers and integrated diagnostic approaches are revolutionizing the field of non-invasive diagnostics in many areas, including the cardiovascular field, allowing ultra-sensitive capture of individual heterogeneity for increasingly personalized medicine [21,22,23,24]. The study population of 75 individuals was categorized into control, obCAD, and non_obCAD groups, and several clinical features were assessed. These groupings were made to investigate differences in clinical features among individuals with varying degrees of coronary artery disease. The study highlighted the significance of age, Agatston score, and hypertension related to coronary artery disease. One of the key findings of the study was that age and Agatston variables showed significant differences among the groups. Both obCAD and non_obCAD groups exhibited significantly higher age and Agatston scores when compared to the control group (CTRL). Additionally, obCAD participants had significantly higher Agatston values compared to those with non_obCAD. Agatston values, which are derived from coronary computed tomography angiography (CCTA), are often used as an indicator of coronary artery calcification and disease severity. Another noteworthy result was the statistically significant association with hypertension. The study found that hypertension was more prevalent among individuals with obCAD and non_obCAD compared to the control group. This suggests a link between hypertension and coronary artery disease, which is consistent with existing medical knowledge. Indeed, hypertension can cause damage to the endothelium, triggering an inflammatory response, and inflamed endothelial cells are less capable of regulating blood flow and clotting, which can lead to the formation of atherosclerotic plaques in the coronary arteries. Furthermore, hypertension is often associated with increased oxidative stress in the blood vessels, and this oxidative stress can promote inflammation and contribute to the progression of CAD. Hypertension is linked to the release of pro-inflammatory mediators in the body. These mediators can exacerbate the inflammatory processes involved in CAD. High blood pressure places increased stress on the heart, which can lead to left ventricular hypertrophy and ultimately heart failure. This stress can also exacerbate the inflammatory response in the coronary arteries.

Individuals with hypertension are at a higher risk of developing CAD due to these interconnected mechanisms. This risk is often compounded when combined with other risk factors like high cholesterol, smoking, and diabetes [25]. In the analysis of coronary artery plaques, this study found that the main coronary vessel affected was the intermediate branch of the left anterior descending artery (IVA) for both non_obCAD and obCAD groups. Furthermore, plaque characteristics were investigated. Approximately 52.38% of cases exhibited calcified components in their plaques, while 28.57% had predominantly non-calcified plaques. The remaining cases featured a mixed component, including both calcified and non-calcified components, with a roughly equal distribution between the obCAD and non_obCAD groups. One of the critical findings of this study was that the Agatston score had a significant predictive effect in discriminating between obCAD and non_obCAD. The odds ratio (OR) was 1.004, with a 95% confidence interval (CI) ranging from 1.002 to 1.007, and a p-value of 0.002. This result suggests that the Agatston score derived from CCTA could be a valuable diagnostic tool for differentiating between individuals with obstructive and non-obstructive coronary artery disease.

Previous research highlighted the fundamental role of cytokines and chemokines in coronary atherogenesis, atherosclerotic plaque formation, cardiovascular inflammatory changes, acute coronary thrombosis, and CAD. Several studies showed the association between serum cytokines, in particular IL-6, and CAD severity [26,27]. Others revealed that serum MCP-1 levels were associated with coronary artery disease, as measured by the coronary artery calcium score [28], and increased plasma levels of IL-6 and TNF-alpha were associated with left ventricular diastolic dysfunction in patients with stable coronary artery disease [29]. In accordance with the literature, in this study, we revealed a close association between serum cytokines and severe CAD. Patients in the CAD group showed an increase in sRAGE, CCL2_MCP1, FLT1, and IL6 levels compared to controls. The cytokines sCD40L, FLT1, sRAGE, CCL2-MCP1, and TNFα showed a gradually increasing trend going towards a more severe form of the obCAD disease. Moreover, utilizing one-way ANOVA, we observed significant differences in cytokine levels among the three patient groups: CTRL, obCAD, and non_obCAD. Specifically, non_obCAD patients demonstrated increased CCL2_MCP1 and IL6 levels compared to controls, whereas obCAD patients showed elevated CCL2_MCP1 and FLT1 levels in comparison.

The investigation into the correlation between clinical features and cytokine concentrations unveiled noteworthy findings. There were significant negative correlations between sST2 and height, BSA and creatinine, IL10 and creatinine, IL18 and HDL, IL6 and creatinine, and LIGHT and glycemia and systolic pressure. Additionally, the CTRL group displayed a significant negative correlation between IL18 and HDL, along with a significant positive correlation between IL18 and triglycerides.

To further assess their predictive capabilities for severe CAD among patients suspected of having CAD, we conducted ROC curve analysis. The results reaffirmed the prognostic significance of IL6 in distinguishing between CTRL and non_obCAD, while CCL2_MCP1 and FLT1 exhibited a substantial discriminatory accuracy between CTRL and obCAD.

**Figure 5:**
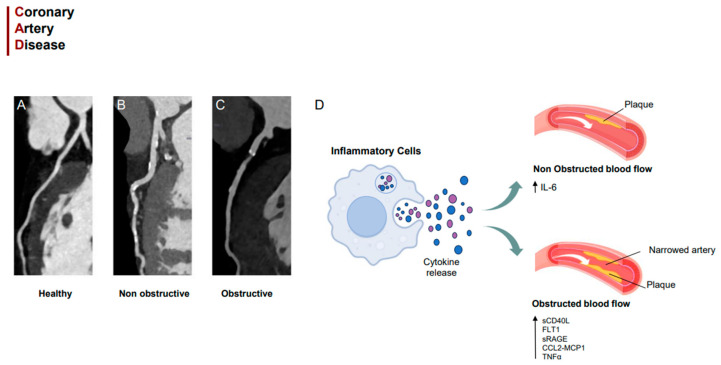
Grafical abstract, (**A**) healthy artery, (**B**) non-obstructive artery, (**C**) obstructive artery, (**D**) grafical illustration.

Additionally, through univariate logistic regression analysis, we delved into the individual performance of the cytokines in predicting CAD diagnosis. The findings indicated that higher IL6 levels increase the odds of being non_obCAD-positive, while elevated CCL2-MCP1 or FLT1 levels raise the odds of being obCAD-positive (Figure 5).

Our study has some limitations: for a better population screening, we chose only high-quality CCTA acquisitions with technological improvements and high spatial and temporal resolution. This resulted in a reduced sample size compared with the high quality of the images and the wide range of data obtainable from CCTA. This includes the Agatston score, selection of patients with non-obstructive CAD (therefore non-significant stenosis), and obstructive CAD with stenosis assessment greater than 50%. Regarding future prospects, the possibility to process images with dedicated software by defining the composition of individual atherosclerotic plaques should be considered [30].

In conclusion, our study provides critical insights into the intricate relationship between serum cytokines and severe CAD. These findings may contribute to guiding the risk stratification and pave the way for novel approaches in identifying and managing patients with advanced CAD, ultimately contributing to improved patient outcomes and a more effective battle against this life-threatening disease. Indeed, these findings add valuable insights into the role of cytokines in CAD progression and possibly establish a set of biomarkers that could aid in identifying patients at greater risk of severe coronary artery lesions. This information could lead to earlier interventions and more targeted treatment strategies for individuals diagnosed with CAD.

## Figures and Tables

**Table 1:** Sample size for each cytokine and group of the population enrolled (*n* = 75).

Citochine	Non-Obstructive CAD (*n* = 34)	Obstructive CAD (*n* = 21)	Controls (*n* = 20)
CCL2_MCP1	34	21	20
Flt1	12	16	19
IL10	32	20	20
IL18	34	21	20
IL6	27	19	20
LIGHT	25	19	16
PlGF	20	16	12
sCD40L	34	21	20
sRAGE	34	21	20
sST2	17	15	18
TNFalfa	13	19	20

**Table 2:** Clinical features of the population enrolled (*n* = 75).

Clinical Variable	CTRL (*n* = 20)	Non_obCAD (*n* = 34)	obCAD (*n* = 21)	Overall *p*-Value
Sex: female/male	10/10 (*n* = 20)	11/23 (*n* = 34)	5/16 (*n* = 21)	0.197
Familiarity: yes/no	16/4 (*n* = 20)	26/8 (*n* = 34)	14/7 (*n* = 21)	0.586
Smoking: yes/no	5/15 (*n* = 20)	14/20 (*n* = 34)	7/14 (*n* = 21)	0.478
Age	50 [24; 59] (*n* = 20)	64 [46; 75] (*n* = 34)	72 [58; 86] (*n* = 21)	*<0.001*
Weight (Kg)	83.5 [56; 130] (*n* = 20)	78 [46; 130] (*n* = 34)	81 [55; 127] (*n* = 21)	0.387
Height (m)	1.7 [1.5; 1.8] (*n* = 20)	1.7 [1.5; 1.9] (*n* = 34)	1.7 [1.5; 1.8] (*n* = 21)	0.872
Obesity: yes/no	8/12 (*n* = 20)	7/27 (*n* = 34)	7/14 (*n* = 21)	0.284
BMI (Kg/m^2^)	29.5 [22.4; 40.1] (*n* = 20)	26.5 [19.1; 38.8] (*n* = 34)	28.4 [22; 40.9] (*n* = 21)	0.211
BSA (m^2^)	1.9 [1.6; 2.5] (*n* = 20)	1.9 [1.4; 2.5] (*n* = 34)	1.9 [1.5; 2.4] (*n* = 21)	0.621
Diabetes: yes/no	4/16 (*n* = 20)	5/29 (*n* = 34)	3/18 (*n* = 21)	0.849
Glycemia (mg/dL)	94.5 [72; 121] (*n* = 10)	97 [78; 228] (*n* = 22)	94.5 [80; 145] (*n* = 14)	0.613
Hypertension: yes/no	15/5 (*n* = 20)	25/9 (*n* = 34)	21/0 (*n* = 21)	*0.017*
Systolic Pressure (mmHg)	126 [115; 170] (*n* = 11)	120 [110; 180] (*n* = 11)	130 [120; 160] (*n* = 14)	0.589
Diastolic Pressure (mmHg)	85 [70; 110] (*n* = 11)	80 [70; 100] (*n* = 11)	80 [70; 110] (*n* = 14)	0.760
Cholesterol: yes/no	14/6 (*n* = 20)	24/10 (*n* = 34)	15/6 (*n* = 21)	0.995
Cholesterol (mg/dL)	179 [149; 245] (*n* = 12)	180.5 [94; 222] (*n* = 20)	173 [121; 286] (*n* = 17)	0.457
HDL (mg/dL)	53 [32; 75] (*n* = 11)	45 [35; 55] (*n* = 15)	42.5 [20; 87] (*n* = 16)	0.322
LDL (mg/dL)	144.5 [63; 168] (*n* = 8)	102 [40; 148] (*n* = 13)	98 [70; 162] (*n* = 11)	0.151
Triglycerides: yes/no	14/6 (*n* = 20)	26/8 (*n* = 34)	15/6 (*n* = 21)	0.851
Triglycerides (mg/dL)	117.5 [53; 218] (*n* = 10)	108 [49; 223] (*n* = 16)	164 [64; 274] (*n* = 15)	0.254
Creatinine (mg/dL)	0.9 [0.5; 1.3] (*n* = 19)	0.9 [0.6; 1.5] (*n* = 26)	1 [0.7; 2] (*n* = 21)	0.507
FC (%)	74.5 [51; 112] (*n* = 20)	69 [43; 104] (*n* = 33)	67 [41; 100] (*n* = 21)	0.190
Agatston	0 [0; 0] (*n* = 20)	54.5 [1; 1238] (*n* = 34)	477 [8; 2942] (*n* = 21)	*<0.001*

**Table 3:** Serum concentrations (pg/mL) and statistical significance of cytokines in 3 groups (CTRL, obCAD, non_obCAD).

Cytokines	CTRL (*n*)	Non_obCAD (*n*)	obCAD (*n*)	Overall *p*-Value	CTRL vs. Non_obCAD	CTRL vs. obCAD	obCAD vs. Non_obCAD
CCL2_MCP1	21.1 [9; 53.3] (*n* = 20)	35 [8.1; 74.5] (*n* = 34)	40.9 [15.2; 253.2] (*n* = 21)	*0.001*	*0.023*	*0.001*	0.540
Flt1	383 [119.1; 2347] (*n* = 19)	1557.1 [50.1; 2827.1] (*n* = 12)	948.3 [334.6; 10053.8] (*n* =16)	*0.003*	0.060	*0.003*	1
IL6	2 [0.4; 25.7] (*n* = 20)	8 [0.7; 32.2] (*n* = 27)	4.5 [0.9; 41.5] (*n* = 19)	*0.006*	*0.004*	0.203	0.702
IL10	1.3 [0.7; 19.1] (*n* = 20)	1.8 [0.7; 40.2] (*n* = 32)	1.4 [0.7; 4.6] (*n* = 20)	0.098			
IL18	462.2 [210.5; 937.6] (*n* = 20)	465.6 [15.9; 999.9] (*n* = 34)	590.7 [337.4; 1919] (*n* = 21)	0.062			
LIGHT	24 [8.8; 157.7] (*n* = 16)	15.3 [3; 91.6] (*n* = 25)	22.1 [9.3; 776.8] (*n* = 19)	0.066			
PlGF	118.2 [30.2; 764] (*n* = 12)	68.9 [22.1; 632] (*n* = 20)	76.7 [30.4; 745.1] (*n* = 16)	0.645			
sCD40L	574.6 [230.8; 1081.8] (*n* = 20)	581.6 [117.7; 21017.7] (*n* = 34)	445.2 [228.4; 22302.2] (*n* = 21)	0.529			
sRAGE	317.1 [88.6; 807.1] (*n* = 20)	556.8 [106; 1664.1] (*n* = 34)	430 [90.7; 2879.5] (*n* = 21)	0.052			
sST2	405.2 [150.2; 1744.7] (*n* = 18)	663 [144.8; 1655.8] (*n* = 17)	381.7 [135.7; 1050] (*n* = 15)	0.156			
TNFalfa	12.9 [1.9; 55.2] (*n* = 20)	13.5 [5.6; 136.2] (*n* = 13)	12.1 [4.5; 107.3] (*n* = 19)	0.686			

**Table 4:** Spearman correlation matrix between cytokine concentration and clinical variables for each group (CTRL, obCAD, non_obCAD).

Cytokines	Clinical Variables	CTRL			Non_obCAD			obCAD		
		*n*	r	p	*n*	r	p	*n*	r	p
sST2	Height	18	−0.2	0.412	17	0.1	0.683	15	−0.6	*0.029*
	BSA	18	0	0.951	17	0.1	0.765	15	−0.6	*0.02*
	Creatinine	17	0	0.993	14	0.3	0.358	15	−0.7	0.007
IL10	Weight	20	−0.6	*0.004*	32	0	0.868	20	−0.5	0.048
	BSA	20	−0.7	*0.002*	32	0.1	0.739	20	−0.5	0.045
	Creatinine	19	−0.2	0.391	25	0	0.854	20	−0.7	*<0.001*
IL18	HDL	11	−0.7	*0.01*	15	−0.1	0.864	16	−0.7	*0.001*
	Triglycerides	10	0.6	0.06	16	0.3	0.233	15	0.6	*0.02*
IL6	Creatinine	19	0.2	0.391	20	−0.1	0.64	19	−0.6	*0.013*
LIGHT	Glycemia	9	−0.1	0.798	17	−0.3	0.291	13	−0.6	*0.022*
	Systolic pressure	8	−0.3	0.482	9	0.5	0.226	13	−0.7	*0.016*
CCL2_MCP1	HDL	11	−0.7	*0.03*	15	−0.2	0.589	16	−0.5	0.069
	Triglycerides	10	0.7	*0.016*	16	−0.1	0.858	15	0.2	0.524
sCD40L	Cholesterol	12	0.7	*0.013*	20	0.2	0.409	17	−0.3	0.254

**Table 5:** Univariate logistic regression analysis of cytokines associated with non_obCAD and obCAD.

Cytokines	Diagnosis	OR	95% CI	*p*-Value
CCL2_MCP1	obCAD	1.08	1.03–1.16	*0.009*
Flt1	obCAD	1.00	1.00–1.01	*0.036*
IL6	non_obCAD	1.15	1.03–1.35	*0.042*

## Data Availability

The original contributions presented in the study are included in the article, further inquiries can be directed to the corresponding author.

## Abbreviation

CAD, coronary artery disease; CCL2_MCP1, CC-motif ligand 2- monocyte chemoattractant protein-1; CCTA, through coronary computed tomography angiography; HF, heart failure; IL10, interleukin 10; IL18, interleukin 18; IL-1β, interleukin 1beta; IL6, interleukin 6; non_obCAD, non-obstructive CAD; obCAD, obstructive CAD; PIGF, placental-like growth factor; RAGE, receptor for advanced glycation end products; sCD40L, soluble CD40 ligand; sFlt-1, soluble Fms-like tyrosine kinase-1; sRAGE, soluble advanced glycation end products; sST2, soluble suppression of tumorigenicity 2; TNFR1, tumor necrosis factor receptor; TNF-α, tumor necrosis factor.
